# Prognostic differences between pre-existing atrial fibrillation in chronic kidney disease and new-onset atrial fibrillation at hemodialysis initiation: a retrospective single-center cohort study

**DOI:** 10.1371/journal.pone.0320336

**Published:** 2025-03-25

**Authors:** Tomohisa Tsuyuki, Mineaki Kitamura, Haruka Fukuda, Takuma Ishii, Kenta Torigoe, Hiroshi Yamashita, Takahiro Takazono, Noriho Sakamoto, Hiroshi Mukae, Tomoya Nishino

**Affiliations:** 1 Department of Nephrology, Nagasaki University Graduate School of Biomedical Sciences, Nagasaki, Japan; 2 Department of Nephrology, Nagasaki Harbor Medical Center, Nagasaki, Japan; 3 Department of Nephrology, Japan Red Cross Nagasaki Genbaku Hospital, Nagasaki, Japan; 4 Department of Respiratory Medicine, Nagasaki University Graduate School of Biomedical Sciences, Nagasaki, Japan; AdventHealth Daytona Beach, UNITED STATES OF AMERICA

## Abstract

Atrial fibrillation (AF) can develop in patients with chronic kidney disease. However, the impact of new-onset AF in patients who are initiated on hemodialysis remains unclear. We categorized 254 patients who were started on hemodialysis into three groups: those with pre-existing AF, those with new-onset AF, and those without AF. Statistical analyses were performed to evaluate the associations between patient characteristics and survival outcomes. AF was observed in 42 patients (16.5%), of whom 19 (7.5%) had pre-existing AF and 23 (9.1%) developed new-onset AF at the initiation of hemodialysis. Multivariate logistic regression models showed that only low serum albumin levels were associated with AF (P = 0.04). Age- and other factors-adjusted multivariable Cox regression models indicated that AF, particularly pre-existing AF, was an independent risk factor for death after dialysis initiation (hazard ratio [HR]: 2.28, 95% confidence interval [CI]: 1.39–3.74, P = 0.001; HR: 3.05, 95% CI: 1.64–5.66, P = 0.004, respectively). However, new-onset AF was not significantly associated with mortality (HR: 1.43, 95% CI: 0.74–2.78, P = 0.28). These findings suggest that pre-existing AF before hemodialysis initiation has a crucial impact on patient prognosis.

## Introduction

Atrial fibrillation (AF), an arrhythmia caused by irregular electrical activity in the atria, is commonly encountered in clinical practice. AF poses significant social and medical challenges, with a reported prevalence of approximately 1% in the general population [1]. This condition is even more prevalent among patients with end-stage kidney disease (ESKD) and those undergoing hemodialysis, with rates ranging from 3.8% to 27% [2,3]. In this population, AF is associated with cerebrovascular disease and all-cause mortality [4]. Additionally, studies indicate that patients with chronic kidney disease (CKD) have an increased risk of developing AF [5]. Patients with both AF and CKD exhibit higher rates of cerebrovascular events and all-cause mortality than those without these conditions [6]. Therefore, AF that develops in non-dialysis-dependent CKD (NDD-CKD) and AF that develops after initiating hemodialysis are both linked to poor prognoses.

Vazquez et al. reported a 12.1% prevalence of AF at the initiation of dialysis, including peritoneal dialysis [7]. Similarly, Tanaka et al. found a prevalence of 6.1% of AF at the initiation of dialysis in 17 Japanese institutions [8]. Furthermore, before the initiation of hemodialysis, hemodynamic instability is more common due to increased circulating blood volume and arteriovenous fistula (AVF) formation, which increases the likelihood of AF onset. Some studies have also documented the prevalence of new-onset AF in patients undergoing renal replacement therapy due to acute kidney injury, ranging from 14% to 37% [9,10]. However, limited research has addressed the differences in all-cause mortality following dialysis initiation between patients with pre-existing AF and those with new-onset AF at the start of hemodialysis. This study aimed to investigate the effects of pre-existing and new-onset AF on all-cause mortality in patients with ESKD initiating hemodialysis. Additionally, we explored factors associated with AF development during this critical phase.

## Methods

### Study population

Patients who underwent hemodialysis at the Nephrology Department of Nagasaki Harbor Medical Center between 2016 and 2023 were included in this study. Dialysis was typically initiated when symptoms such as pulmonary edema or uremia were observed. If congestive heart failure was present in ESKD, diuretics were initiated first, and dialysis was initiated if the patient failed to improve. Dialysis was also initiated for uremia when symptoms such as general malaise and anorexia became pronounced. Additionally, as the Nagasaki Harbor Medical Center is an emergency hospital, some patients with NDD-CKD had deteriorated renal function due to serious illness (e.g., infection or acute myocardial infarction) requiring urgent dialysis. Hemodialysis was initiated by providing an AVF. If an AVF was unavailable, a central venous catheter was inserted into the internal jugular or femoral vein.

Almost all patients initiating dialysis underwent an electrocardiogram at our institution before dialysis induction. Electrocardiogram data at the time of dialysis initiation were based on recordings collected within three months prior to dialysis induction in accordance with a previous study [11]. Patients without electrocardiogram data in their electronic medical records within the 3-month period were excluded from the analysis. In addition, patients aged < 18 years or who opted for peritoneal dialysis were excluded. AF was defined using the ICD-10 (International Classification of Diseases, 10th Revision) code I48. AF on electrocardiography is characterized by 1) irregular R-R intervals, 2) the absence of distinct, repeating P waves, and 3) irregular atrial activity [12]. Paroxysmal AF is defined as AF that terminates spontaneously or with intervention within 7 days of onset. Persistent AF is defined as AF that persists for ≥7 days [12]. Newly developing AF during the hospitalization which the hemodialysis was initiated was defined as new-onset AF, whereas AF present in patients with NDD-CKD was defined as pre-existing AF.

The study was approved by the Nagasaki Harbor Medical Center Institutional Review Board (IRB) under approval number R05-30 and conformed to the ethical standards outlined in the 1964 Declaration of Helsinki and its subsequent revisions. The need for informed consent was waived due to the retrospective nature of the study and the anonymized data collection.

### Data collection

Patient data were collected at the time of the first referral to our institution and initiation of hemodialysis. The presence of comorbidities, history of smoking, prescriptions, and medical history were confirmed at the initiation of hemodialysis. In addition, the causes, such as infection, uremia, congestive heart failure, or ischemic heart disease, that led to the initiation of dialysis were collected from the electronic medical records. Patient prognosis following hemodialysis initiation was monitored until February 2024 in collaboration with maintenance dialysis centers in Nagasaki City.

### Statistical analysis

The results were presented as percentages and counts for categorical variables and as mean ± standard deviation or median (interquartile range) for continuous variables with non-normal distributions. The chi-square test was used to assess categorical variables, whereas the Wilcoxon rank-sum test or Kruskal–Wallis test was used to assess continuous variables. Bonferroni’s post-hoc analysis was performed for multiple comparisons. Univariate and multivariate logistic regression analyses for AF were performed. Multivariate analyses included variables in the univariate logistic regression analysis. Differences in prognosis between patients with and without AF undergoing hemodialysis were evaluated using log-rank analysis. Additionally, prognostic differences among patients with preexisting AF, new-onset AF, and those without AF were examined. Univariable and multivariable Cox regression analyses were performed. Model 1 focused on AF (preexisting AF and new-onset AF), Model 2 specifically addressed preexisting AF, and Model 3 examined new-onset AF. Each model included the following variables that were significant in the univariable analysis: age, sex, AVF created before hemodialysis initiation, and serum albumin levels at hemodialysis initiation. Statistical significance was set at two-tailed p-values of <0.05. Sensitivity analysis was performed using stratified Cox regression analysis according to age groups (<70, 70–79, and ≥80 years). All statistical analyses were performed using JMP Pro (version 17.0; SAS Institute, Cary, NC, USA).

## Results

### Baseline characteristics

**Fig 1:**
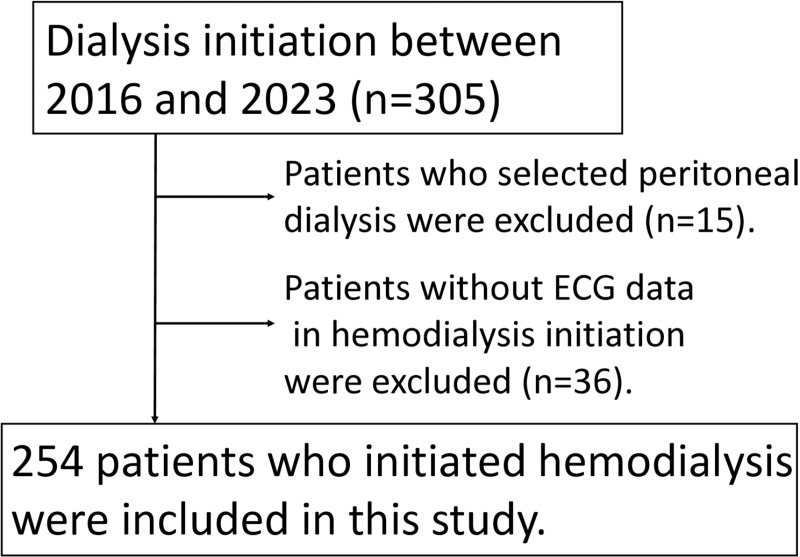
Flowchart of patient selection. Based on the inclusion and exclusion criteria, a total of 254 patients were included in the study.

A total of 305 patients were started on renal replacement therapy, particularly hemodialysis, at our institution between 2016 and 2023. Eighteen patients who were initiated on peritoneal dialysis were excluded from this study. Additionally, 36 patients who did not undergo electrocardiograms during dialysis initiation were excluded. None of the patients were younger than 18 years. Finally, 254 patients were included in this study (Fig 1).

Of these 254 patients, 42 (16.5%) were diagnosed with AF. Of these, 19 (7.5%) patients had pre-existing AF at NDD-CKD, and 23 (9.1%) developed new-onset AF during hemodialysis initiation. New-onset AF occurred at a median (interquartile range (IQR)) of 1 day (IQR: −1 to 5 days) before the commencement of hemodialysis. Among patients with pre-existing AF, four had paroxysmal AF and 15 had persistent AF. Among those with new-onset AF, 13 had paroxysmal AF and 10 had persistent AF. In the pre-existing AF group, 16 patients were on anticoagulant therapy, with a mean prothrombin time-international normalized ratio of 1.61 ± 0.63 at the time of first referral. The median duration from the onset of pre-existing AF to the initiation of dialysis was estimated to be 5.5 (IQR: 1.5 to 24.5) years.

In this study cohort, the average age of patients was 71.9 ±  11.6 years, with male participants accounting for 67.3% of the population. The patients in the AF group (including pre-existing AF and new-onset AF) were significantly older than those in the non-AF group (P = 0.04). The AF group also had a significantly greater history of ischemic heart disease than the non-AF group (P = 0.04). The proportion of patients requiring central venous catheter insertion at hemodialysis initiation was significantly higher in the AF group (P = 0.02). Additionally, a significantly higher proportion of patients presented with infections at the commencement of hemodialysis (P < 0.001), with pneumonia being the most common infection and significantly more prevalent in the AF group than in the non-AF group (P < 0.001). A significantly higher proportion of patients in the AF group developed ischemic heart disease (P = 0.003). Serum albumin levels were significantly lower in the AF group (P = 0.008), whereas serum C-reactive protein levels were significantly higher in the AF group (P < 0.001) (Table 1).

**Table 1:** Clinical characteristics of patients initiating HD with AF and without AF.

	All (n = 254)	AF (n = 42)	Non-AF (n = 212)	P-value
**Age (years)**	71.9 ± 11.6	75.0 ± 10.90	71.3 ± 11.7	**0.04**
**Male (%)**	171 (67.3)	30 (71.4)	141 (66.5)	0.53
**Body mass index (kg/m**^**2**^)	23.4 ± 4.5	22.8 ± 5.4	23.5 ± 4.3	0.12
**Diabetes (%)**	129 (50.8)	21 (50.0)	108 (50.9)	0.91
**Hypertension (%)**	243 (95.7)	39 (92.9)	204 (96.2)	0.33
**Hyperlipidemia (%)**	111 (43.7)	20 (47.6)	91 (42.9)	0.58
**History of smoking (%)**				0.31
** Never smoked**	111 (45.7)	14 (35.0)	97 (47.8)	
** Past smoker**	77 (31.7)	16 (40.0)	61 (30.1)	
** Current smoker**	55 (22.6)	10 (25.0)	45 (22.2)	
** Unknown**	11 (4.3)	2 (4.8)	9 (4.3)	
**Duration from first visit to HD initiation (days)**	349 (135 to 871)	311 (33 to 746)	352 (145 to 892)	0.29
**History of ischemic heart disease (%)**	49 (19.3)	13 (31.0)	36 (16.9)	**0.04**
**History of ischemic stroke (%)**	21 (8.3)	4 (9.5)	17 (8.0)	0.75
**History of cerebral hemorrhage (%)**	5 (2.0)	0 (0)	5 (2.4)	0.31
**CVC insertion at the initiation of HD (%)**	56 (22.1)	15 (35.7)	41 (19.3)	**0.02**
**AVF created before the initiation of HD (%)**	211 (83.1)	30 (71.4)	181 (85.4)	**0.03**
**Any infections at the HD initiation (%)**	46 (18.1)	20 (47.6)	26 (12.3)	**<0.001**
**Pneumonia at the HD initiation (%)**	35 (13.8)	13 (31.0)	22 (10.4)	**0.004**
**Congestive heart failure at the HD initiation (%)**	48 (18.9)	8 (19.1)	40 (18.9)	0.98
**Ischemic heart disease at the HD initiation (%)**	7 (2.8)	4 (9.5)	3 (1.4)	**0.003**
**Blood tests at the initiation of hemodialysis**				
**White blood cell (× 10**^**3**^** **µ **L)**	7.0 ± 3.6	8.2 ± 5.9	6.7 ± 2.9	0.25
**Hemoglobin (g/dL)**	9.0 ± 1.4	9.4 ± 1.5	9.0 ± 1.4	0.09
**Creatinine (mg/dL)**	8.6 ± 3.5	8.1 ± 4.5	8.6 ± 3.3	0.053
**Blood urea nitrogen (mg/dL)**	91.5 ± 29.7	100.4 ± 33.1	89.8 ± 28.7	0.06
**eGFR (mL/min/1.73m**^**2**^)	5.1 ± 2.0	5.4 ± 1.8	5.1 ± 2.1	0.09
**Albumin (g/dL)**	3.1 ± 1.0	2.8 ± 0.6	3.1 ± 0.6	**0.008**
**Potassium (mEq/L)**	4.4 ± 0.8	4.3 ± 0.9	4.4 ± 0.8	0.60
**Corrected calcium (mg/dL)**	8.7 ± 0.9	8.9 ± 0.8	8.7 ± 0.9	**0.02**
**Phosphorus (mg/dL)**	6.2 ± 2.2	5.7 ± 2.0	6.3 ± 2.2	0.10
**Intact PTH (pg/mL)**	235 (153 to 360)	205 (124 to 321)	242 (163 to 362)	0.14
**C-reactive protein (mg/dL)**	0.3 (0.1 to 2.1)	3.5 (0.3 to 12.9)	0.2 (0 to 1.2)	**<0.001**
**Causes of ESKD (%)**				0.67
** Diabetic kidney disease**	114 (44.9)	19 (45.2)	95 (44.8)	
** Nephrosclerosis**	72 (28.4)	11 (26.2)	61 (28.8)	
** Glomerulonephritis**	37 (14.6)	5 (11.9)	32 (15.1)	
** ADPKD**	3 (1.2)	0 (0)	3 (1.4)	
** Others**	28 (11.0)	7 (16.7)	21 (9.9)	
**Prescription at the initiation of HD (%)**				
** RAS inhibitors**	76 (29.9)	9 (21.4)	67 (31.6)	0.19
** Diuretics**	175 (68.9)	30 (71.4)	145 (68.4)	0.70
** Vitamin D**	99 (39.0)	14 (33.3)	85 (40.1)	0.41
** ESA**	172 (67.7)	25 (59.5)	147 (69.3)	0.21
** HIF-PHI**	16 (6.3)	3 (7.1)	13 (6.1)	0.81

AF, atrial fibrillation; HD, hemodialysis; CVC, central venous catheter; AVF, arteriovenous fistula; eGFR, estimated glomerular filtration rate; ESKD, end-stage kidney disease; ADPKD, autosomal dominant polycystic kidney disease; PTH, parathormone; RAS inhibitors, renin angiotensin system inhibitors; ESA, erythropoietin stimulating agents; HIF-PHI, hypoxia-inducible factor prolyl hydroxylase inhibitors.

Analysis of Wilcoxon ranked sum test for quantitative and Pearson’s χ^2^-test for qualitative variables. P < 0.05 was considered statistically significant.

vs Non-AF group P < 0.05.

Patients in the pre-existing AF group were significantly older than those in the non-AF group, and the new-onset AF group had significantly higher rates of pneumonia and ischemic heart disease at the commencement of hemodialysis compared to the non-AF group. Furthermore, the new-onset AF group had significantly higher serum C-reactive protein levels and white blood cell counts than the non-AF group (Table 2).

**Table 2:** Clinical characteristics data of patients initiating HD with pre-existing AF and new-onset AF.

	Pre-existing AF (n = 19)	New-onset AF (n = 23)	Non-AF (n = 212)	P-value
**Age (years)**	78.2 ± 6.2^*^	72.4 ± 13.2	71.3 ± 11.7	**0.04**
**Male (%)**	16 (84.2)	14 (60.8)	141 (66.5)	0.23
**Body mass index (kg/m**^**2**^)	23.5 ± 5.0	22.2 ± 5.8	23.5 ± 4.3	0.19
**Diabetes (%)**	8 (42.1)	13 (56.5)	108 (50.9)	0.64
**Hypertension (%)**	18 (94.7)	21 (91.3)	204 (96.2)	0.53
**Hyperlipidemia (%)**	9 (47.4)	11 (47.8)	91 (42.9)	0.85
**History of smoking (%)**				0.36
** Never smoked**	7 (36.8)	7 (33.3)	97 (47.8)	
** Past smoker**	9 (47.4)	7 (33.3)	61 (30.1)	
** Current smoker**	3 (15.8)	7 (33.3)	45 (22.2)	
** Unknown**	0 (0)	2 (8.7)	9 (4.3)	
**Duration from first visit to HD initiation (days)**	267 (105 to 1478)	369 (16 to 709)	352 (145 to 892)	0.38
**History of ischemic heart disease (%)**	6 (31.6)	7 (30.4)	36 (16.9)	0.11
**History of ischemic stroke (%)**	2 (10.5)	2 (8.7)	17 (8.0)	0.93
**History of cerebral hemorrhage (%)**	0 (0)	0 (0)	5 (2.4)	0.60
**CVC insertion at the initiation of HD (%)**	6 (31.6)	9 (39.1)	41 (19.3)	0.054
**AVF created before the initiation of HD (%)**	14 (73.7)	16 (69.6)	181 (85.4)	0.08
**Any infections at the HD initiation (%)**	6 (31.6)^*^	14 (60.9)^*^	26 (12.3)	**<0.001**
**Pneumonia at the HD initiation (%)**	3 (15.8)	10 (43.5)^*^	22 (10.4)	**<0.001**
**Congestive heart failure at the HD initiation (%)**	6 (31.6)	2 (8.7)	40 (18.9)	0.17
**Ischemic heart disease at the HD initiation (%)**	1 (5.3)	3 (13.0)^*^	3 (1.4)	**0.004**
**Blood tests at the initiation of HD**				
**Creatinine (mg/dL)**	7.8 ± 2.3	8.4 ± 5.7	8.6 ± 3.3	0.14
**Blood urea nitrogen (mg/dL)**	99.2 ± 22.8	101.4 ± 40.2	89.8 ± 28.7	0.14
**eGFR (mL/min/1.73m**^**2**^)	5.2 ± 1.3	5.5 ± 2.1	5.1 ± 2.1	0.23
**Albumin (g/dL)**	2.9 ± 0.6	2.7 ± 0.6^*^	3.1 ± 0.6	**0.02**
**Hemoglobin (g/dL)**	9.6 ± 1.6	9.2 ± 1.5	9.0 ± 1.3	0.19
**Potassium (mEq/L)**	4.3 ± 0.9	4.3 ± 0.8	4.4 ± 0.8	0.84
**Corrected calcium (mg/dL)**	9.0 ± 0.6	8.8 ± 1.0	8.7 ± 0.9	0.07
**Phosphorus (mg/dL)**	5.5 ± 1.5	5.8 ± 2.4	6.3 ± 2.2	0.26
**Intact PTH (pg/mL)**	259 (146 to 386)	176 (91 to 251)	242 (163 to 362)	0.07
**C-reactive protein (mg/dL)**	0.72 (0.1 to 6.2)^*^	5.8 (1 to 18.1)^*^^,^^†^	0.2 (0 to 1.2)	**<0.001**
**White blood cell (×10** ^**3**^ ** µ L)**	6.2 ± 3.4	9.9 ± 6.9^*^^,^^†^	6.7 ± 2.9	**0.006**
**Causes of ESKD (%)**				0.94
** Diabetic kidney disease**	8 (42.1)	11 (47.8)	95 (44.8)	
** Nephrosclerosis**	6 (31.6)	5 (21.7)	61 (28.8)	
** Glomerulonephritis**	2 (10.5)	3 (13.0)	32 (15.1)	
** ADPKD**	0 (0)	0 (0)	3 (1.42)	
** Others**	3 (15.8)	4 (17.4)	21 (9.9)	
**Prescription at the initiation of HD (%)**				
** RAS inhibitors**	6 (31.6)	3 (13.0)	67 (31.6)	0.18
** Diuretics**	16 (84.2)	14 (60.9)	145 (68.4)	0.25
** Vitamin D**	8 (42.1)	6 (26.1)	85 (40.1)	0.41
** ESA**	11 (57.9)	14 (60.9)	147 (69.3)	0.45
** HIF-PHI**	2 (10.5)	1 (4.3)	13 (6.1)	0.69

AF, atrial fibrillation; HD, hemodialysis; CVC, central venous catheter; AVF, arteriovenous fistula; eGFR, estimated glomerular filtration rate; ESKD, end-stage kidney disease; ADPKD, autosomal dominant polycystic kidney disease; PTH, parathormone; RAS inhibitors, renin angiotensin system inhibitors; ESA, erythropoietin stimulating agents; HIF-PHI, hypoxia-inducible factor prolyl hydroxylase inhibitors.

Analysis of Kruskal-Wallis test for quantitative and Pearson’s χ^2^-test for qualitative variables. P < 0.05 was considered statistically significant.

* vs Non-AF group P < 0.05

† vs Pre-existing AF group P < 0.05.

### Factors associated with the presence of AF at the initiation of hemodialysis

Univariate logistic regression analysis revealed that AF was associated with a history of ischemic heart disease, having an AVF created before the initiation of hemodialysis, and albumin levels at the start of hemodialysis. In multivariate analysis, only serum albumin levels were associated with AF (Table 3).

**Table 3:** Univariable and multivariable logistic regression analyses affecting atrial fibrillation at the initiation of hemodialysis.

	Univariable			Multivariable		
	OR	95% CI	P value	OR	95% CI	P value
**Age at the initiation of HD**	1.03	1.00–1.06	0.054			
**Male**	1.26	0.61–2.61	0.54			
**BMI at the initiation of HD**	0.96	0.89–1.04	0.32			
**History of smoking (current or past vs never)**	1.70	0.84–3.44	0.14			
**History of ischemic heart disease**	2.19	1.04–4.62	**0.04**	2.11	0.98–4.54	0.06
**Diabetes Mellitus**	0.96	0.50–1.87	0.91			
**Hypertension**	0.51	0.13–2.01	0.34			
**AVF created before the initiation of HD**	0.43	0.20–0.93	**0.03**	0.55	0.24–1.24	0.15
**eGFR at the initiation of HD**	1.06	0.91–1.24	0.45			
**Serum albumin at the initiation of HD**	0.47	0.27–0.82	**0.008**	0.54	0.31–0.97	**0.04**

OR, odds ratio; CI, confidence interval; HD, hemodialysis; BMI, body mass index; eGFR, estimated glomerular filtration rate; AVF, arteriovenous fistula.

P <  0.05 was considered statistically significant

### Mortality and risk factors for poor outcomes in patients with AF undergoing hemodialysis

**Fig 2:**
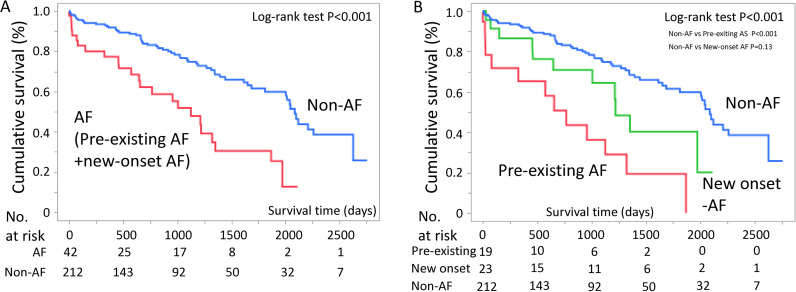
Survival analyses. a) The Kaplan–Meier curve for all-cause mortality after hemodialysis initiation shows that the group with atrial fibrillation had a poorer prognosis than those without atrial fibrillation (P <  0.001). b) The Kaplan–Meier curve compares the pre-existing atrial fibrillation, new-onset atrial fibrillation, and non-atrial fibrillation groups. The log-rank test revealed a significant difference among the three groups (P <  0.001).

The patients were followed up until February 2024, with a median follow-up duration of 786 days (IQR: 330–1401). During the observation period after the initiation of hemodialysis, 85 patients died. Infection was the most common cause of death (n = 29, 34.1%), followed by sudden death (n = 10, 11.8%), heart failure or myocardial infarction (n = 7, 8.2%), cerebral hemorrhage (n = 5, 5.9%), and malignancy (n = 3, 3.5%). Fig 2a shows the Kaplan–Meier curve comparing patients with AF (including new-onset AF and pre-existing AF) and those without AF. The prognosis of patients with AF was significantly worse than that of patients without AF (P <  0.001). Additionally, Fig 2b illustrates differences in survival between the groups with new-onset AF, pre-existing AF, and non-AF.

Univariable and multivariable Cox regression analyses demonstrated that AF and pre-existing AF were associated with increased mortality (Table 4). However, new-onset AF did not contribute to all-cause mortality.

**Table 4:** Univariable and multivariable Cox regression analysis for prognosis after the initiation of hemodialysis.

	Univariable			Model 1			Model 2			Model 3		
	HR	95% CI	P value	HR	95% CI	P value	HR	95% CI	P value	HR	95% CI	P value
**Age at the initiation of HD**	1.07	1.04–1.09	**<0.001**	1.06	1.04–1.09	**<0.001**	1.06	1.04–1.09	**<0.001**	1.07	1.04–1.09	**<0.001**
**Male**	1.68	1.03–2.73	**0.04**	1.90	1.15–3.12	**0.01**	1.87	1.14–3.10	**0.01**	2.00	0.30–0.82	**0.007**
**History of ischemic heart disease**	1.47	0.90–2.39	0.12									
**Diabetes Mellitus**	0.84	0.55–1.29	0.42									
**Hypertension**	1.64	0.40–6.67	0.49									
**History smoking** **(current or past vs never)**	1.18	0.76–1.84	0.47									
**AVF created before the initiation of HD**	0.53	0.31–0.90	**0.02**	0.57	0.31–1.02	0.06	0.52	0.29–0.92	**0.03**	0.50	0.28–0.91	**0.02**
**Any infections at the initiation of HD**	1.55	0.94–2.56	0.09									
**Ischemic heart disease at the initiation of HD**	1.87	0.59–5.98	0.29									
**Albumin at the initiation of HD**	0.48	0.32–0.71	**0.003**	0.61	0.40–0.92	**0.009**	0.56	0.36–0.85	**0.007**	0.62	0.41–0.94	**0.03**
**AF (pre-existing** + **new-onset) at the initiation of HD**	2.77	1.71–4.48	**<0.001**	2.28	1.39–3.74	**0.001**						
**Pre-existing AF at the initiation of HD**	4.14	2.27–7.56	**<0.001**				3.05	1.64–5.66	**<0.001**			
**New-onset AF at the initiation of HD**	1.62	0.86–3.07	0.14							1.43	0.74–2.78	0.28

HR, hazard ratio; CI, confidence interval; HD, hemodialysis; AVF, arteriovenous fistula; AF, atrial fibrillation

P < 0.05 was considered statistically significant.

**Fig 3:**
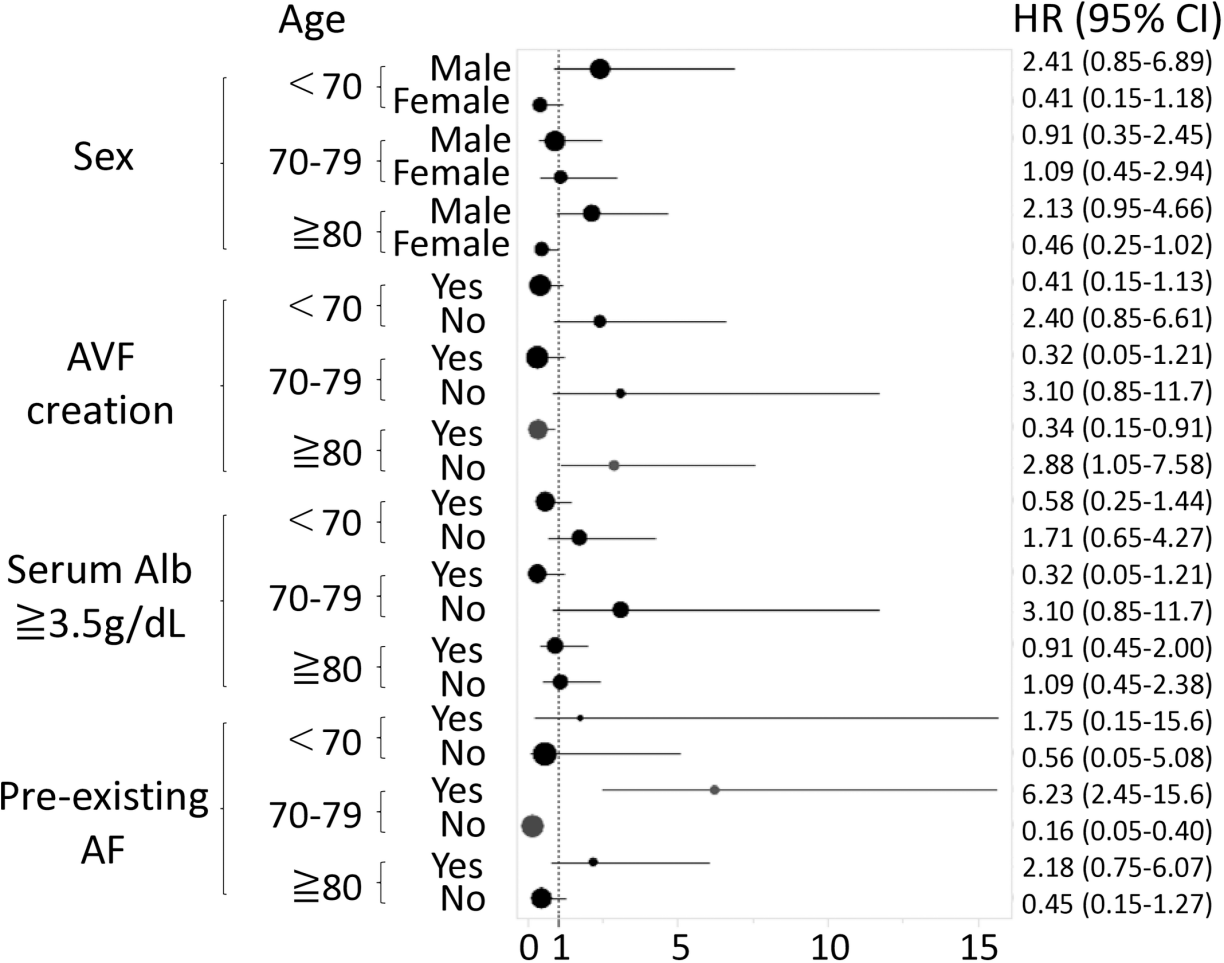
Stratified Cox regression analysis for patient survival. AF: atrial fibrillation, Alb: albumin, AVF: arteriovenous fistula, CI: confidence interval, HR: hazard ratio. Each error bar represents the range from the lower to the upper confidence limit. Patients were stratified into three age groups (<70, 70–79, and ≥ 80 years). In the stratified multivariable Cox regression analysis, pre-existing AF was significantly associated with prognosis in patients aged 70–79 years.

Sensitivity analysis findings in relation to stratified Cox regression according to age groups (<70, 70–79, and ≥ 0 years) showed that pre-existing AF had the greatest effect on increased mortality in the 70–79 age group, and this effect did not worsen with increasing age (Fig 3).

## Discussion

In this single-center retrospective cohort study, we demonstrated the prognostic significance of AF at hemodialysis initiation and investigated the differences in prognosis between pre-existing AF and new-onset AF. Our findings suggest that pre-existing AF in patients with NDD-CKD may result in worse outcomes than new-onset AF at the initiation of hemodialysis or in patients without AF. Furthermore, low serum albumin levels at the initiation of hemodialysis were associated with an increased risk of developing AF. To our knowledge, few studies have compared pre-existing AF with new-onset AF in demonstrating this association.

AF is frequently observed in patients undergoing hemodialysis. Roy et al. reported that paroxysmal AF can be detected in as many as 41% of individuals receiving maintenance hemodialysis using an implanted loop recorder [13]. AF can also be present at the time of dialysis initiation; Vazquez et al. reported AF in 31 of 256 patients (12.1%) at the start of dialysis [7], and Tanaka et al. reported AF in 93 patients (6.1%) at the start of dialysis among 1516 patients [8]. In the present study, AF was present in 16.5% of patients, which is slightly higher than previously reported rates. The prevalence of AF generally increases with age, including in patients undergoing hemodialysis [14,15]. For this reason, this study included a larger proportion of older adults than the other two studies. By contrast, new-onset AF can occur in patients undergoing renal replacement therapy under serious conditions [9]. Hellman et al. reported that among 516 patients requiring renal replacement therapy, 190 (37%) developed new-onset AF in the intensive care unit [10]. Although the incidence of new-onset AF was low in this study (9.1%), it may be attributable to the inclusion of patients with less severe disease at the initiation of dialysis.

Risk factors for the development of AF without ESKD include older age, history of heart failure, hypertension, and diabetes mellitus [15,16]. In patients with CKD, including those with ESKD, low kidney function, albuminuria, and AVF creation before hemodialysis are strongly associated with the incidence of AF, independent of other risk factors [5,17]. Recent studies have also reported that low blood albumin levels are an independent risk factor for AF development. They suggested that decreased serum albumin levels may be linked to reductions in anti-inflammatory and antioxidant capacities, potentially leading to atrial structural and electrical remodeling, which in turn could promote the development of AF [18]. In this study, we found that low serum albumin levels were independently associated with AF in patients initiating hemodialysis.

Some reports have shown that AF is associated with higher all-cause mortality and worse cardiovascular outcomes [19,20]. Additionally, patients with AF at the start of dialysis have been found to have a higher mortality rate than those without AF [7,8]. Melissa et al. reported that persistent AF, compared to paroxysmal AF upon first diagnosis, is independently associated with increased mortality in patients with non-valvular AF [21]. Prolonged exposure to AF induces various pathophysiological changes in the atria that further promote remodeling by enlarging the atria and decreasing atrial contractility [22]. In this study, all-cause mortality was significantly worse for AF observed at the time of NDD-CKD than for AF at the start of hemodialysis. In contrast, new-onset AF did not affect mortality. Three possible explanations for this result are as follows. We consider the lack of a significant effect of new-onset AF on mortality likely to be because of the small sample size, which limited our ability to detect a significant difference between patients with new-onset AF and those without AF. In addition, fluid overload might cause temporary AF. After the initiation of hemodialysis, fluid balance may be restored. Finally, some patients developed infections, such as pneumonia, during the initiation of hemodialysis. Since infections can induce tachycardia, AF may occur as a result. Once the symptoms have improved, AF may resolve.

Numerous studies have proposed radiofrequency catheter ablation as a promising technique for treating AF in patients not undergoing hemodialysis [23]. However, reports on the outcomes of catheter ablation for AF in patients undergoing hemodialysis are scarce. The sinus rhythm maintenance rate after the first catheter ablation in patients undergoing hemodialysis was likely lower than that in patients not undergoing hemodialysis, which is consistent with previous findings [24]. Patients undergoing hemodialysis often have a larger atrial diameter, possibly because of fluid retention, which is an important predictor of poor prognosis after catheter ablation for AF [25]. In addition, AF development is associated with poor progression of renal function [26]. Therefore, treating AF early in its development, especially at the NDD-CKD stage, may improve outcomes.

This study has certain limitations. First, this was a single-center study with a small sample size in Nagasaki City, which limited the generalizability of the results. Second, anticoagulant agents, including warfarin, were discontinued in our hospital after the commencement of hemodialysis; however, it is unknown whether they may have been resumed at a maintenance dialysis facility. Third, unmeasured factors that may have been associated with prognosis were not considered in this analysis.

AF was found to have a significant impact on mortality in patients undergoing hemodialysis, especially when AF develops during the NDD-CKD phase. Furthermore, low serum albumin levels were found to be associated with the development of AF. Early interventions, such as radiofrequency catheter ablation at the NDD-CKD stage, could improve mortality in patients undergoing maintenance hemodialysis.

## Supporting Information

S1 Data Patients’ data are supplied in a supporting information file. (XLSX)
