# Supplementary material for: Prognostic differences between pre-existing atrial fibrillation in chronic kidney disease and new-onset atrial fibrillation at hemodialysis initiation: a retrospective single-center cohort study
Source: PLoS One. 2025 Mar 25;20(3):e0320336. doi: 10.1371/journal.pone.0320336 (PMC11936237; doi:10.1371/journal.pone.0320336)
Supplement: Attachment — Submitted filename: Response 0208.docx [file pone.0320336.s003.docx]

Dear Editors and Reviewers,

We are grateful for providing your time and effort to review our manuscript and provide valuable feedback, which we believe has enhanced its overall quality. Below, we have addressed all of your concerns and indicated the page and line numbers of the respective changes. We hope that the revisions and our responses will be found satisfactory. Nevertheless, we remain open to any further suggestions for improving the manuscript.

Editor's comments –

Response: Thank you for reviewing the paper; we appreciate your valuable comments.

1. Lines 80-82 mention, "Newly developing AF during the hospitalization period following hemodialysis initiation was defined as new-onset AF, whereas AF present in patients with NDD-CKD was defined as pre-existing AF. Line 127 says, "New-onset AF occurred at a median (interquartile range (IQR)) of 1 day (IQR: -1 to 5 days) before the commencement of hemodialysis". Why were these people categorized into the new-onset A fib if they developed A fib 1-5 days before the initiation of HD? These two sentences are somewhat contradictory. You might need to redefine new-onset AFib if you want to include patients who developed atrial fibrillation during the hospitalization during which the hemodialysis was initiated rather than saying, 'developing atrial fibrillation following hemodialysis initiation'.

Response: Thank you for your important opinion. In accordance with your suggestions, we have defined New-onset AF as “the development of AF during the hospitalization for hemodialysis initiation.” (pages 5-6, lines 80-82).

2. It is surprising that new-onset atrial fibrillation did not contribute to all-cause mortality in your study. Could you please explain why these results were noted in your study and add the reasons for them in the discussion?

Response: Thank you for your important question. There are a few possibilities to explain this phenomenon. First, the number of included patients would be small, which would not be suitable for detecting the effect of AF during the hospitalization for hemodialysis initiation. Second, fluid overload might cause the AF temporarily. After hemodialysis initiation, the fluid balance might be restored. Third, some patients developed infections, such as pneumonia, during the hemodialysis initiation. As infections can induce tachycardia, AF might be accompanied. After improving the symptoms, AF would be resolved. We have added this speculation in the discussion (pages 17-18, lines 248-254).

3. We know that the mortality of ESRD patients increases consistently with the increased age of patients at dialysis initiation. Is it possible that much higher age, which was noted in patients with pre-existing atrial fibrillation, could have contributed to their increased mortality on top of A fib?

Response: Thank you for your valuable question. We were concerned that this finding might be associated with increasing age. Therefore, we used stratified Cox regression analysis, with patients divided into three age groups (<70 years, 70–79 years, and ≥80 years). Figure 3 shows that pre-existing AF was significantly associated with prognosis in patients aged 70–79 years. (page14, lines 204-206).

Thank you again for your valuable comments. We hope the revised manuscript has been improved to a quality worthy of publication in the PLOS one.

Reviewer1 comments

The article is written is fluent English, the research question is very valid and has clinical implications as A.fib has high prevalence in CKD patients.

Could not find lot of literature on outcomes of pre-existing A.fib vs new A fib in CKD patients.

The study provided valuable results and information such as focusing on hypoalbuminemia to prevent A.fib.

Study provides future direction to evaluate for A.fib ablation in NDD-CKD stage to improve mortality.

Although the statistical analysis are performed correctly, the sample size is small to conclude that pre-existing A.fib has higher mortality than new onset A.fib. Same is true for unknown confounders and anticoagulation statuss.

Nonetheless the study offers valuable insights and needs validation by larger multi-center studies.

Response: Thank you for reviewing our paper; we greatly appreciate your time and effort in evaluating our manuscript. We agree that more extensive study is needed, and this is also discussed in the limitations section.

Reviewer2 comments

This is a retrospective, small, single center study looking at the prognosis of patients with pre-existing A fib in CKD vs new development of A fib at dialysis initiation vs no A fib. It showed statistical difference with poor prognosis in patients with pre-existing A fib.

- It is well written study. Has some limitations

- Increasing the power of the study would be helpful.

Response: Thank you for reviewing our paper greatly appreciate the time and effort you put into evaluating our manuscript and your valuable comments. As you mentioned, the number of the included patients would be too small to draw rigid conclusions. However, our results will be informative in clinical practice.

1. What is the difference of A fib in this population compared to general population with respect to common risks factors? Most of A fib does increase with age and so is your population under study

Response: Thank you for your important question. We believe that, compared to general risk factors, CKD particularly in the period before dialysis initiation poses a greater risk for AF development. Similar to our findings, Vazquez et al. and Tanaka et al. reported a significant increase in the frequency of AF during the induction phase of dialysis [7, 8].

2. Do we know at what stage was A fib diagnosed in pre-existing A fib group? Was it diagnosed early in CKD vs advanced CKD? Was it long standing CKD and was it well controlled and managed?

Response: Thank you for your important question. Among patients with pre-existing AF, four had paroxysmal AF, and 15 had persistent AF. Sixteen patients were on anticoagulants, with a mean prothrombin time-international normalized ratio of 1.61 ± 0.63 at the time of the first referral. The median duration from the onset of pre-existing AF to the initiation of dialysis was estimated to be 5.5 years (IQR: 1.5 to 24.5 years) (page 8, lines 128–132). Based on this, we speculate that AF might not have been adequately controlled due to improper management of anticoagulant therapy. We could not determine whether AF was diagnosed in the early stages of CKD or in advanced CKD, as it was not noted in the electronic medical records.

3. Would be good to include younger patients with A fib to see how would that change prognosis? Was pre-existing A fib--were there any paroxysmal cases or all of them had persistent A fib? which can help to know if paroxysmal A fib does contribute to increased mortality. Does that need to be managed aggressively as well?

Response: Thank you for your insightful question. As you pointed out, we speculate that age plays a crucial role in prognosis in patients with or without AF. Therefore, we conducted a stratified Cox regression analysis, which showed that pre-existing AF was significantly associated with prognosis in patients aged 70–79 years (page 14, lines 2204–206). Generally, catheter ablation has been widely used to treat AF and has shown better efficacy for paroxysmal AF than for persistent AF. In our study, pre-existing AF included some patients with paroxysmal AF. Based on these findings, we propose that an earlier-stage intervention may be beneficial.

Thank you again for your valuable comments. We hope the revised manuscript has been improved to a quality worthy of publication in the PLOS one.
