# Supplementary material for: Prognostic differences between pre-existing atrial fibrillation in chronic kidney disease and new-onset atrial fibrillation at hemodialysis initiation: a retrospective single-center cohort study
Source: PLoS One. 2025 Mar 25;20(3):e0320336. doi: 10.1371/journal.pone.0320336 (PMC11936237; doi:10.1371/journal.pone.0320336)
Supplement: Peer review history

# Author comment: Author response to Decision Letter 0

**DOI:** 10.1371/journal.pone.0320336.r001

27 Dec 2024

# Peer review: Decision Letter 0

**DOI:** 10.1371/journal.pone.0320336.r002

**Authors:** Prathap kumar Simhadri

21 Jan 2025

PONE-D-24-59770Prognostic differences between pre-existing atrial fibrillation in chronic kidney disease and new-onset atrial fibrillation at hemodialysis initiation: a retrospective single-center cohort study

PLOS ONE

Dear Dr. Kitamura,

Thank you for submitting your manuscript to PLOS ONE. After careful consideration, we feel that it has merit but does not fully meet PLOS ONE’s publication criteria as it currently stands. Therefore, we invite you to submit a revised version of the manuscript that addresses the points raised during the review process.

==============================

Dear authors,

Thank you for submitting interesting manuscript. The question this study tried to address is a valid one and it adds value to the current literature. This manuscript needs to be significantly revised before it could be considered for acceptance.  Please submit a revised manuscript after addressing the questions raised by both the editor and the reviewers. 

Editor's comments -

Lines 80-82 mention, "Newly developing AF during the hospitalization period following hemodialysis initiation was defined as new-onset AF, whereas AF present in patients with NDD-CKD was defined as pre-existing AF. Line 127 says, "New-onset AF occurred at a median (interquartile range (IQR)) of 1 day (IQR: -1 to 5 days) before the commencement of hemodialysis".  Why were these people categorized into the new-onset A fib if they developed A fib 1-5 days before the initiation of HD? These two sentences are somewhat contradictory. You might need to redefine new-onset AFib if you want to include patients who developed atrial fibrillation during the hospitalization during which the hemodialysis was initiated rather than saying, 'developing atrial fibrillation following hemodialysis initiation'. It is surprising that new-onset atrial fibrillation did not contribute to all-cause mortality in your study. Could you please explain why these results were noted in your study and add the reasons for them in the discussion? We know that the mortality of ESRD patients increases consistently with the increased age of patients at dialysis initiation. Is it possible that much higher age, which was noted in patients with pre-existing atrial fibrillation, could have contributed to their increased mortality on top of A fib?Please ensure that your decision is justified on PLOS ONE’s publication criteria  and not, for example, on novelty or perceived impact.

==============================

Please submit your revised manuscript by Mar 07 2025 11:59PM. If you will need more time than this to complete your revisions, please reply to this message or contact the journal office at plosone@plos.org . When you're ready to submit your revision, log on to https://www.editorialmanager.com/pone/ and select the 'Submissions Needing Revision' folder to locate your manuscript file.

Please include the following items when submitting your revised manuscript:

- A rebuttal letter that responds to each point raised by the academic editor and reviewer(s). You should upload this letter as a separate file labeled 'Response to Reviewers'.
- A marked-up copy of your manuscript that highlights changes made to the original version. You should upload this as a separate file labeled 'Revised Manuscript with Track Changes'.
- An unmarked version of your revised paper without tracked changes. You should upload this as a separate file labeled 'Manuscript'.

If you would like to make changes to your financial disclosure, please include your updated statement in your cover letter. Guidelines for resubmitting your figure files are available below the reviewer comments at the end of this letter.

If applicable, we recommend that you deposit your laboratory protocols in protocols.io to enhance the reproducibility of your results. Protocols.io assigns your protocol its own identifier (DOI) so that it can be cited independently in the future. For instructions see: https://journals.plos.org/plosone/s/submission-guidelines#loc-laboratory-protocols . Additionally, PLOS ONE offers an option for publishing peer-reviewed Lab Protocol articles, which describe protocols hosted on protocols.io. Read more information on sharing protocols at https://plos.org/protocols?utm_medium=editorial-email&utm_source=authorletters&utm_campaign=protocols .

We look forward to receiving your revised manuscript.

Kind regards,

Prathap kumar Simhadri, MD

Academic Editor

PLOS ONE

Journal Requirements:

When submitting your revision, we need you to address these additional requirements.

1. Please ensure that your manuscript meets PLOS ONE's style requirements, including those for file naming. The PLOS ONE style templates can be found at 

https://journals.plos.org/plosone/s/file?id=wjVg/PLOSOne_formatting_sample_main_body.pdf and 

https://journals.plos.org/plosone/s/file?id=ba62/PLOSOne_formatting_sample_title_authors_affiliations.pdf

2. Please remove all personal information, ensure that the data shared are in accordance with participant consent, and re-upload a fully anonymized data set. 

Note: spreadsheet columns with personal information must be removed and not hidden as all hidden columns will appear in the published file.

Additional guidance on preparing raw data for publication can be found in our Data Policy (https://journals.plos.org/plosone/s/data-availability#loc-human-research-participant-data-and-other-sensitive-data) and in the following article: http://www.bmj.com/content/340/bmj.c181.long.

[Note: HTML markup is below. Please do not edit.]

Reviewers' comments:

Reviewer's Responses to Questions

**Comments to the Author**

1. Is the manuscript technically sound, and do the data support the conclusions?

The manuscript must describe a technically sound piece of scientific research with data that supports the conclusions. Experiments must have been conducted rigorously, with appropriate controls, replication, and sample sizes. The conclusions must be drawn appropriately based on the data presented. 

Reviewer #1: Yes

Reviewer #2: Yes

**********

2. Has the statistical analysis been performed appropriately and rigorously? 

Reviewer #1: Yes

Reviewer #2: I Don't Know

**********

3. Have the authors made all data underlying the findings in their manuscript fully available?

The PLOS Data policy requires authors to make all data underlying the findings described in their manuscript fully available without restriction, with rare exception (please refer to the Data Availability Statement in the manuscript PDF file). The data should be provided as part of the manuscript or its supporting information, or deposited to a public repository. For example, in addition to summary statistics, the data points behind means, medians and variance measures should be available. If there are restrictions on publicly sharing data—e.g. participant privacy or use of data from a third party—those must be specified.

Reviewer #1: Yes

Reviewer #2: Yes

**********

4. Is the manuscript presented in an intelligible fashion and written in standard English?

PLOS ONE does not copyedit accepted manuscripts, so the language in submitted articles must be clear, correct, and unambiguous. Any typographical or grammatical errors should be corrected at revision, so please note any specific errors here.

Reviewer #1: Yes

Reviewer #2: Yes

**********

5. Review Comments to the Author

Please use the space provided to explain your answers to the questions above. You may also include additional comments for the author, including concerns about dual publication, research ethics, or publication ethics. (Please upload your review as an attachment if it exceeds 20,000 characters)

Reviewer #1: The article is written is fluent English, the research question is very valid and has clinical implications as A.fib has high prevalence in CKD patients.

Could not find lot of literature on outcomes of pre-existing A.fib vs new A fib in CKD patients.

The study provided valuable results and information such as focusing on hypoalbuminemia to prevent A.fib.

Study provides future direction to evaluate for A.fib ablation in NDD-CKD stage to improve mortality.

Although the statistical analysis are performed correctly, the sample size is small to conclude that pre-existing A.fib has higher mortality than new onset A.fib. Same is true for unknown confounders and anticoagulation statuss.

Nonetheless the study offers valuable insights and needs validation by larger multi-center studies.

Reviewer #2: This is a retrospective, small, single center study looking at the prognosis of patients with pre-existing A fib in CKD vs new development of A fib at dialysis initiation vs no A fib. It showed statistical difference with poor prognosis in patients with pre-existing A fib.

- It is well written study. Has some limitations

- Increasing the power of the study would be helpful.

- What is the difference of A fib in this population compared to general population with respect to common risks factors? Most of A fib does increase with age and so is your population under study

- Do we know at what stage was A fib diagnosed in pre-existing A fib group? Was it diagnosed early in CKD vs advanced CKD? Was it long standing CKD and was it well controlled and managed?

- Would be good to include younger patients with A fib to see how would that change prognosis ?

- Was pre-existing A fib--were there any paroxysmal cases or all of them had persistent A fib? which can help to know if paroxysmal A fib does contribute to increased mortality. Does that need to be managed aggressively as well?

- what were the duration of A fb in pre-existing population? was it well controlled? were they well controlled?

**********

6. PLOS authors have the option to publish the peer review history of their article (what does this mean? ). If published, this will include your full peer review and any attached files.

If you choose “no”, your identity will remain anonymous but your review may still be made public.

**Do you want your identity to be public for this peer review?** For information about this choice, including consent withdrawal, please see our Privacy Policy .

Reviewer #1: **Yes: ** Babu Sriram Maringanti

Reviewer #2: No

**********

[NOTE: If reviewer comments were submitted as an attachment file, they will be attached to this email and accessible via the submission site. Please log into your account, locate the manuscript record, and check for the action link "View Attachments". If this link does not appear, there are no attachment files.]

While revising your submission, please upload your figure files to the Preflight Analysis and Conversion Engine (PACE) digital diagnostic tool, https://pacev2.apexcovantage.com/ . PACE helps ensure that figures meet PLOS requirements. To use PACE, you must first register as a user. Registration is free. Then, login and navigate to the UPLOAD tab, where you will find detailed instructions on how to use the tool. If you encounter any issues or have any questions when using PACE, please email PLOS at figures@plos.org . Please note that Supporting Information files do not need this step.

# Author comment: Author response to Decision Letter 1

**DOI:** 10.1371/journal.pone.0320336.r003

7 Feb 2025

Dear Editors and Reviewers,

We are grateful for providing your time and effort to review our manuscript and provide valuable feedback, which we believe has enhanced its overall quality. Below, we have addressed all of your concerns and indicated the page and line numbers of the respective changes. We hope that the revisions and our responses will be found satisfactory. Nevertheless, we remain open to any further suggestions for improving the manuscript.

Editor's comments –

Response: Thank you for reviewing the paper; we appreciate your valuable comments.

1. Lines 80-82 mention, "Newly developing AF during the hospitalization period following hemodialysis initiation was defined as new-onset AF, whereas AF present in patients with NDD-CKD was defined as pre-existing AF. Line 127 says, "New-onset AF occurred at a median (interquartile range (IQR)) of 1 day (IQR: -1 to 5 days) before the commencement of hemodialysis". Why were these people categorized into the new-onset A fib if they developed A fib 1-5 days before the initiation of HD? These two sentences are somewhat contradictory. You might need to redefine new-onset AFib if you want to include patients who developed atrial fibrillation during the hospitalization during which the hemodialysis was initiated rather than saying, 'developing atrial fibrillation following hemodialysis initiation'.

Response: Thank you for your important opinion. In accordance with your suggestions, we have defined New-onset AF as “the development of AF during the hospitalization for hemodialysis initiation.” (pages 5-6, lines 80-82).

2. It is surprising that new-onset atrial fibrillation did not contribute to all-cause mortality in your study. Could you please explain why these results were noted in your study and add the reasons for them in the discussion?

Response: Thank you for your important question. There are a few possibilities to explain this phenomenon. First, the number of included patients would be small, which would not be suitable for detecting the effect of AF during the hospitalization for hemodialysis initiation. Second, fluid overload might cause the AF temporarily. After hemodialysis initiation, the fluid balance might be restored. Third, some patients developed infections, such as pneumonia, during the hemodialysis initiation. As infections can induce tachycardia, AF might be accompanied. After improving the symptoms, AF would be resolved. We have added this speculation in the discussion (pages 17-18, lines 248-254).

3. We know that the mortality of ESRD patients increases consistently with the increased age of patients at dialysis initiation. Is it possible that much higher age, which was noted in patients with pre-existing atrial fibrillation, could have contributed to their increased mortality on top of A fib?

Response: Thank you for your valuable question. We were concerned that this finding might be associated with increasing age. Therefore, we used stratified Cox regression analysis, with patients divided into three age groups (<70 years, 70–79 years, and ≥80 years). Figure 3 shows that pre-existing AF was significantly associated with prognosis in patients aged 70–79 years. (page14, lines 204-206).

Thank you again for your valuable comments. We hope the revised manuscript has been improved to a quality worthy of publication in the PLOS one.

Reviewer1 comments

The article is written is fluent English, the research question is very valid and has clinical implications as A.fib has high prevalence in CKD patients.

Could not find lot of literature on outcomes of pre-existing A.fib vs new A fib in CKD patients.

The study provided valuable results and information such as focusing on hypoalbuminemia to prevent A.fib.

Study provides future direction to evaluate for A.fib ablation in NDD-CKD stage to improve mortality.

Although the statistical analysis are performed correctly, the sample size is small to conclude that pre-existing A.fib has higher mortality than new onset A.fib. Same is true for unknown confounders and anticoagulation statuss.

Nonetheless the study offers valuable insights and needs validation by larger multi-center studies.

Response: Thank you for reviewing our paper; we greatly appreciate your time and effort in evaluating our manuscript. We agree that more extensive study is needed, and this is also discussed in the limitations section.

Reviewer2 comments

This is a retrospective, small, single center study looking at the prognosis of patients with pre-existing A fib in CKD vs new development of A fib at dialysis initiation vs no A fib. It showed statistical difference with poor prognosis in patients with pre-existing A fib.

- It is well written study. Has some limitations

- Increasing the power of the study would be helpful.

Response: Thank you for reviewing our paper greatly appreciate the time and effort you put into evaluating our manuscript and your valuable comments. As you mentioned, the number of the included patients would be too small to draw rigid conclusions. However, our results will be informative in clinical practice.

1. What is the difference of A fib in this population compared to general population with respect to common risks factors? Most of A fib does increase with age and so is your population under study

Response: Thank you for your important question. We believe that, compared to general risk factors, CKD particularly in the period before dialysis initiation poses a greater risk for AF development. Similar to our findings, Vazquez et al. and Tanaka et al. reported a significant increase in the frequency of AF during the induction phase of dialysis [7, 8].

2. Do we know at what stage was A fib diagnosed in pre-existing A fib group? Was it diagnosed early in CKD vs advanced CKD? Was it long standing CKD and was it well controlled and managed?

Response: Thank you for your important question. Among patients with pre-existing AF, four had paroxysmal AF, and 15 had persistent AF. Sixteen patients were on anticoagulants, with a mean prothrombin time-international normalized ratio of 1.61 ± 0.63 at the time of the first referral. The median duration from the onset of pre-existing AF to the initiation of dialysis was estimated to be 5.5 years (IQR: 1.5 to 24.5 years) (page 8, lines 128–132). Based on this, we speculate that AF might not have been adequately controlled due to improper management of anticoagulant therapy. We could not determine whether AF was diagnosed in the early stages of CKD or in advanced CKD, as it was not noted in the electronic medical records.

3. Would be good to include younger patients with A fib to see how would that change prognosis? Was pre-existing A fib--were there any paroxysmal cases or all of them had persistent A fib? which can help to know if paroxysmal A fib does contribute to increased mortality. Does that need to be managed aggressively as well?

Response: Thank you for your insightful question. As you pointed out, we speculate that age plays a crucial role in prognosis in patients with or without AF. Therefore, we conducted a stratified Cox regression analysis, which showed that pre-existing AF was significantly associated with prognosis in patients aged 70–79 years (page 14, lines 2204–206). Generally, catheter ablation has been widely used to treat AF and has shown better efficacy for paroxysmal AF than for persistent AF. In our study, pre-existing AF included some patients with paroxysmal AF. Based on these findings, we propose that an earlier-stage intervention may be beneficial.

Thank you again for your valuable comments. We hope the revised manuscript has been improved to a quality worthy of publication in the PLOS one.

Attachment Submitted filename: Response 0208.docx

# Editor report: Decision Letter 1

**DOI:** 10.1371/journal.pone.0320336.r004

**Authors:** Prathap kumar Simhadri

18 Feb 2025

Prognostic differences between pre-existing atrial fibrillation in chronic kidney disease and new-onset atrial fibrillation at hemodialysis initiation: a retrospective single-center cohort study

PONE-D-24-59770R1

Dear Dr. Kitamura,

We’re pleased to inform you that your manuscript has been judged scientifically suitable for publication and will be formally accepted for publication once it meets all outstanding technical requirements.

Within one week, you’ll receive an e-mail detailing the required amendments. When these have been addressed, you’ll receive a formal acceptance letter and your manuscript will be scheduled for publication.

An invoice will be generated when your article is formally accepted. Please note, if your institution has a publishing partnership with PLOS and your article meets the relevant criteria, all or part of your publication costs will be covered. Please make sure your user information is up-to-date by logging into Editorial Manager at Editorial Manager®  and clicking the ‘Update My Information' link at the top of the page. If you have any questions relating to publication charges, please contact our Author Billing department directly at authorbilling@plos.org.

If your institution or institutions have a press office, please notify them about your upcoming paper to help maximize its impact. If they’ll be preparing press materials, please inform our press team as soon as possible -- no later than 48 hours after receiving the formal acceptance. Your manuscript will remain under strict press embargo until 2 pm Eastern Time on the date of publication. For more information, please contact onepress@plos.org.

Kind regards,

Prathap kumar Simhadri, MD

Academic Editor

PLOS ONE

Additional Editor Comments (optional):

Thank you very much for addressing all the reviewer comments and submitting the revised manuscript. I believe this study adds value to the current literature.

Reviewers' comments:

# Editor report: Acceptance letter

**DOI:** 10.1371/journal.pone.0320336.r005

**Authors:** Prathap kumar Simhadri

PONE-D-24-59770R1

PLOS ONE

Dear Dr. Kitamura,

I'm pleased to inform you that your manuscript has been deemed suitable for publication in PLOS ONE. Congratulations! Your manuscript is now being handed over to our production team.

At this stage, our production department will prepare your paper for publication. This includes ensuring the following:

* All references, tables, and figures are properly cited

* All relevant supporting information is included in the manuscript submission,

* There are no issues that prevent the paper from being properly typeset

If revisions are needed, the production department will contact you directly to resolve them. If no revisions are needed, you will receive an email when the publication date has been set. At this time, we do not offer pre-publication proofs to authors during production of the accepted work. Please keep in mind that we are working through a large volume of accepted articles, so please give us a few weeks to review your paper and let you know the next and final steps.

Lastly, if your institution or institutions have a press office, please let them know about your upcoming paper now to help maximize its impact. If they'll be preparing press materials, please inform our press team within the next 48 hours. Your manuscript will remain under strict press embargo until 2 pm Eastern Time on the date of publication. For more information, please contact onepress@plos.org.

If we can help with anything else, please email us at customercare@plos.org.

Thank you for submitting your work to PLOS ONE and supporting open access.

Kind regards,

PLOS ONE Editorial Office Staff

on behalf of

Dr. Prathap kumar Simhadri

Academic Editor

PLOS ONE
